# Morphological Divergence Driven by Predation Environment within and between Species of *Brachyrhaphis* Fishes

**DOI:** 10.1371/journal.pone.0090274

**Published:** 2014-02-26

**Authors:** Spencer J. Ingley, Eric J. Billman, Mark C. Belk, Jerald B. Johnson

**Affiliations:** 1 Department of Biology, Brigham Young University, Provo, Utah, United States of America; 2 Monte L. Bean Life Science Museum, Brigham Young University, Provo, Utah, United States of America; University of Basel, Switzerland

## Abstract

Natural selection often results in profound differences in body shape among populations from divergent selective environments. Predation is a well-studied driver of divergence, with predators having a strong effect on the evolution of prey body shape, especially for traits related to escape behavior. Comparative studies, both at the population level and between species, show that the presence or absence of predators can alter prey morphology. Although this pattern is well documented in various species or population pairs, few studies have tested for similar patterns of body shape evolution at multiple stages of divergence within a taxonomic group. Here, we examine morphological divergence associated with predation environment in the livebearing fish genus *Brachyrhaphis*. We compare differences in body shape between populations of *B. rhabdophora* from different predation environments to differences in body shape between *B. roseni* and *B. terrabensis* (sister species) from predator and predator free habitats, respectively. We found that in each lineage, shape differed between predation environments, consistent with the hypothesis that locomotor function is optimized for either steady swimming (predator free) or escape behavior (predator). Although differences in body shape were greatest between *B. roseni* and *B. terrabensis*, we found that much of the total morphological diversification between these species had already been achieved within *B. rhabdophora* (29% in females and 47% in males). Interestingly, at both levels of divergence we found that early in ontogenetic development, females differed in shape between predation environments; however, as females matured, their body shapes converged on a similar phenotype, likely due to the constraints of pregnancy. Finally, we found that body shape varies with body size in a similar way, regardless of predation environment, in each lineage. Our findings are important because they provide evidence that the same source of selection can drive similar phenotypic divergence independently at multiple divergence levels.

## Introduction

Numerous studies have documented adaptation to divergent natural selection regimes [1]–[8]. However, most studies examining fine-scale evolutionary diversification are limited to either between species or within species differences, and as a result, fail to adequately address how the same source of selection drives phenotypic divergence at varying taxonomic levels (a broad but general exception being studies of convergent and parallel evolution). Indeed, few studies have looked at the evolution of adaptive strategies across a speciation continuum (i.e., both within and between species) with the intent of determining how much diversification takes place across different stages of speciation [9]–[11]. The paucity of such studies may be due to the difficulty of identifying systems where similarly divergent selection regimes have driven or are driving divergence at multiple taxonomic levels. These studies are valuable to our understanding of evolutionary diversification, and can help explain how predictable phenotypic divergence is when populations or species are subject to similar selective environments.

Predation has been a focal mechanism of divergent selection since Darwin outlined his theory of evolution by natural selection [12]; indeed, Darwin saw predation-prey interactions as some of the clearest cases of natural selection, and cited numerous examples of adaptation in both predator and prey [12]. Predation is known to affect numerous traits in both predator and prey, including behavior, life history, and morphology [7], [8], [13]–[25]. Morphological adaptations resulting from different predation environments are of particular importance because they reflect both behavioral and life-history adaptations, and such adaptations have been observed in numerous and diverse taxa [8], [20], [26]–[36]. Predators can have a profound effect on the evolution of prey body shape, especially for traits related to escape behavior [37]. Comparative studies of taxa from different ‘predation environments,’ both between populations within species and between species pairs, show a strong link between the presence of predators and overall prey morphology [13], [20], [31]–[36].

Livebearing fishes (Poeciliidae) have been used as model systems in a diversity of ecological and evolutionary studies [6], [23], [38]–[45]. Many of these studies have focused on adaptation to divergent predation environments, specifically examining life-history evolution and morphological divergence driven in large part by the presence or absence of predators [6], [21], [46]–[52]. The live-bearing fish genus *Brachyrhaphis* has become an important model for studying the evolution of predator-mediated adaptations [6], [13], [23], [46]. *Brachyrhaphis* occur primarily in lower Central America (LCA), with many species endemic to Costa Rica and Panama. Several species of *Brachyrhaphis* exhibit adaptation to divergent predation environments, including changes to life-history [46] and morphology [6], [13]. *Brachyrhaphis rhabdophora*, for example, has evolved divergent life-history strategies associated with predation environment that are similar to those observed in numerous other poeciliid species [46], [53]. Studies of adaptation in *Brachyrhaphis* have so far focused exclusively on intra-specific variation, where populations of a given species occur in either ‘predator free’ or ‘predator’ environments. Interestingly, similar patterns of morphological divergence may be present at deeper phylogenetic levels within *Brachyrhaphis* (i.e., between sister species rather than populations within a species; see below). If this is the case, then *Brachyrhaphis* would provide an ideal model system for studying morphological variation both among populations and between species from divergent predation environments, and testing for similar patterns of divergence among different phylogenetic levels to determine how similar selective regimes drive phenotypic divergence.

*Brachyrhaphis roseni* and *B. terrabensis* are sister species [54] that have similar distributions, occurring from southeastern Costa Rica to central Panama along the Pacific versant [55]. Although these species frequently occur within the same drainages, *B. terrabensis* typically occupies higher elevation headwater streams, while *B. roseni* occupies lower elevation coastal streams [55]. Consequently, *B. terrabensis* occurs in streams that are primarily void of piscivorous predators, while *B. roseni* co-occurs with numerous and abundant predators (e.g., *Hoplias microlepis*). This pattern is similar to that observed among populations *within* other poeciliid species [13], [21], [23], [27], [47], [50], [51], including the well-studied sister species to this species pair, *B. rhabdophora* [24], [25], [43], [46], [56]. However, *B. roseni* and *B. terrabensis* are unique because they themselves do not span both predator and predator free environments, but rather are segregated into predator and predator free environments, respectively (Belk et al. in review; unpublished data). Furthermore, *Brachyrhaphis roseni* and *B. terrabensis* have evolved similarly divergent life histories (Belk et al. in review) to those observed between populations of *B. rhabdophora* [46], *B. episcopi* [23], and other poeciliids [21], namely smaller size at maturity with more and smaller offspring in predator environments than in predator free environments. The hypothesis that these species are sister taxa, and the fact that they occur in divergent predation environments and display predictable patterns of life-history divergence, suggests that the selective forces driving divergence between populations of *B. rhabdophora* (i.e., predator vs. predator free environments) might also have driven divergence between *B. roseni* and *B. terrabensis*. This provides an opportunity to compare morphological variation both within (recently diverged) and between species of *Brachyrhaphis* from opposing predation environments in two closely related evolutionary lineages. In addition to testing for gross differences in prey morphology associated with predation environment, our data set allows us to test for similar patterns of morphological divergence both between sexes and among size classes.

In this study, we use geometric morphometric analyses to test four hypotheses related to morphological divergence driven by predation environment in three species of *Brachyrhaphis* fishes. We focus on contrasts between *B. roseni* and *B. terrabensis* and between populations of *B. rhabdophora* from divergent predation environments. Our hypotheses are as follows.

First, we predict that body shape differs between *B. roseni* and *B. terrabensis*, and between populations of *B. rhabdophora* from different predation environments. We predict that populations from predator environments (*B. roseni* and predator *B. rhabdophora*) will be more streamlined and have a more robust caudal peduncle region than populations from predator free environments (*B. terrabensis* and predator free *B. rhabdophora*) due to morphological optimization for different swimming modes [8], [49], [57]–[62]. Co-occurrence with predators should favor the evolution of a body form optimized for fast-start swimming (i.e., greater burst speed ability), needed to evade predator strikes [8]. In contrast, increased resource competition often associated with predator free environments should favor the evolution of a body form optimized for more efficient prolonged swimming, important for finding and consuming food, acquiring mates, and conserving energy for reproduction [8], [49]. Given that these two swimming types are optimized by different propulsor arrangements (i.e., fin size and shape, muscle size and shape), optimizing body shape for one swimming mode necessarily compromises the other. Prolonged swimming performance is optimized with a relatively shallow caudal peduncle, and a deep anterior body/head region. Fast-start swimming is optimized by the opposite trait values, including deep caudal peduncle and a shallow anterior body/head [8], [49], [57]–[62].

Second, we expect to find similar, but more pronounced (i.e., greater magnitude), morphological divergence occurs between sister taxa *Brachyrhaphis roseni* and *B. terrabensis* than occurs between populations of *B. rhabdophora* from different predation environments. This hypothesis focuses on determining how much divergence occurs between populations of *B. rhabdophora* from different predation environments versus between sister species *B. roseni* and *B. terrabensis* from different predation environments. We predict that divergence in body shape between *B. roseni* and *B. terrabensis* will be associated with predation environments as predicted by theory, and that these differences will be similar but more exaggerated than those observed between populations of *B. rhabdophora.* This difference in magnitude could be attributed to several factors, including for example a greater time since divergence or differences in the balance between strength of divergent selection and homogenizing gene flow.

Third, we predict that body shape will vary between sexes, both for the among-species and among-population comparisons. Although the pattern of variation described above is predicted to occur between populations from different predation environments due to divergent natural selection, it is also likely that, within populations, these morphological traits are affected by differences in reproductive roles between sexes, mating strategies among size classes, and ontogenetic changes. Given that *Brachyrhaphis* are live-bearing, females of all three species may be constrained morphologically by pregnancy in the same way [37]. Therefore, we test if patterns of sexual dimorphism show equal magnitude and direction of divergence between contrasting selective environments, essentially addressing the question, do differences in male and female reproductive roles constrain or magnify shape responses to variation in predation environment? We predict that female body shape will converge between predation environments relative to males due to the constraint of pregnancy.

Finally, we test the hypothesis that body shape differs among size classes across predation environments. This hypothesis tests for an interaction between size and species, and addresses potential differences in reproductive roles, alternative-mating strategies among size classes, and ontogenetic effects. We predict that shape will not vary consistently across sizes (i.e., as individuals mature and grow) because of the potential for variation in male reproductive strategy across size classes in *Brachyrhaphis* (i.e., coercive mating versus coaxing), and differences in female reproductive allocation at different sizes.

## Materials and Methods

### Molecular Laboratory Methods and Analysis of Genetic Distance

A primary purpose of this study is to determine how body shape evolves at different phylogenetic levels of divergence (i.e., within and between species) when populations are subject to similarly divergent selective regimes. Although a previous study of *Brachyrhaphis rhabdophora* indicated little molecular divergence among populations from different predation environments [43], the amount of molecular divergence among populations of *B. rhabdophora* compared to the amount of divergence between sister species *B. roseni* and *B. terrabensis* remains relatively unexplored (but see Mojica et al. 1997). Thus, we generated mitochondrial DNA sequences from the cytochrome *b* (cyt*b*) gene for four representative populations of *B. rhabdophora* from different predation environments and for six populations of *B. roseni* and *B. terrabensis* (Table S2). We isolated DNA using the Qiagen DNeasy96 tissue protocol (QIAGEN Sciences, Maryland, USA) and amplified cyt*b* fragments for each sample by PCR, using forward primer GLU31 [63] and reverse primer HD15680 [64]. We followed [65] for amplification and sequencing reactions, clean up, and sequence visualization. We assembled contigs and checked amino acid coding for errors (stop codons) while viewing electropherograms in Geneious [66], and manually aligned sequences in Mesquite v. 2.75 [67]. We obtained a total of 26 *B. rhabdophora*, 16 *B. roseni*, and 18 *B. terrabensis* sequences of a cyt*b* fragment 1140 bp in length (plus ∼65 bp of the downstream gene) representing four, three, and three populations, respectively (Table S2). All sequences were deposited on Genbank under accession numbers KJ081551– KJ081609.

In order to test for varying levels of molecular divergence within and among species of *Brachyrhaphis*, we computed pairwise genetic distances using MEGA5 [68]. We first computed raw pairwise genetic distance. Next, we used a model selection framework (AIC, [69]) within jModelTest 2 [70] to determine the best-fit model of molecular evolution for our data set. We then calculated model-corrected pairwise genetic distances using the best-fit model, TrN+G [71], with the Tamura-Nei model and gamma distributed rates among sites in MEGA5 [68]. Our results show that *B. roseni* and *B. terrabensis* show a greater level of genetic divergence than populations of *B. rhabdophora* from different predation environments. Pairwise population comparisons of cyt*b* among populations of *B. rhabdophora* from different predation environments revealed remarkably little variation (mean model corrected pairwise genetic distance  = 0.004; Table S3). On the contrary, pairwise population comparisons between *B. roseni* and *B. terrabensis* showed genetic distance an order of magnitude greater (mean model corrected pairwise genetic distance  = 0.04; Table S4). Thus, with an expanded sampling both in terms of numbers of base pairs and sequences, we find strong evidence that supports the findings of Johnson (2001) and refute the findings of Mojica et al. (1997). Collectively, these data validate our comparison as one consisting of two levels of phylogenetic divergence.

### Study Sites and Characterizing Predation Environment

**Figure 1:**
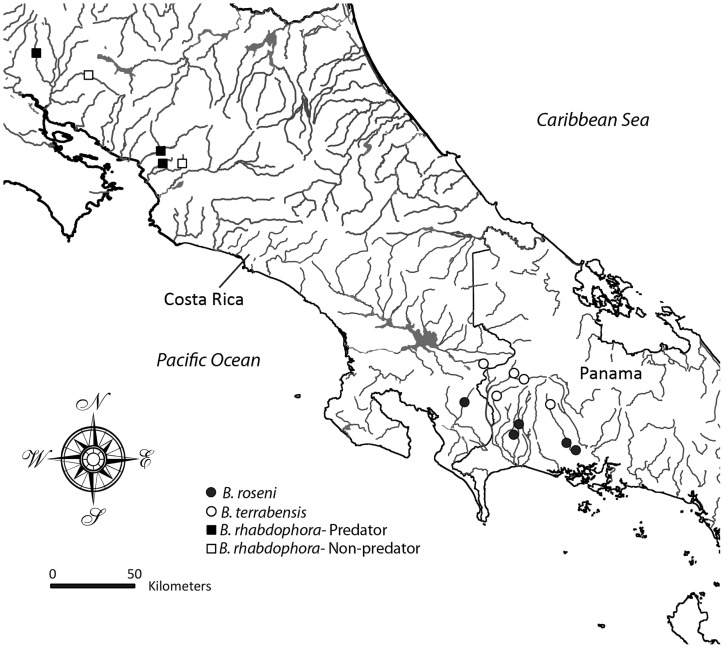
Map of collection sites for *Brachyrhaphis terrabensis*, *B. roseni*, and *B. rhabdophora* used in this study. *Brachyrhaphis terrabensis* (open circles) occur at higher elevations in streams that are void of fish predators. *Brachyrhaphis roseni* (closed circles) occur at lower elevations in streams that have abundant predators. *Brachyrhaphis rhabdophora* occur at sites that are both predator (closed squares) and predation free (open squares).

We collected *Brachyrhaphis roseni* and *B. terrabensis* with a handheld seine from eight streams in the Chiriquí province of Panama between 20 and 29 August 2011, and one population of each species from eastern Costa Rica during 2007 (Figure 1; Table S1). We collected *Brachyrhaphis rhabdophora* from two predator free and three predator environments in Guanacaste region of Costa Rica between 5 and 12 May 2006 (Table S1). All animal collecting was conducted under Brigham Young University IACUC committee approval. All necessary permits were obtained for the described field studies, and no collecting took place on private or protected lands. Collecting and export permits were provided by the Autoridad Nacional del Ambiente in Panama and under the Costa Rican Ministerio del Ambiente y Energía Sistema Nacional de Areas de Conservasión in Costa Rica.

The streams are characterized by a pool-riffle-pool structure, similar to that observed in other *Brachyrhaphis* species [25]. A primary environmental indicator of *B. roseni, B. terrabensis*, and *B. rhabdophora* life history divergence is the presence or absence of piscivorous predators (e.g., *Parachromis dovii* and *Hoplias microlepis* [24], [25], [46], unpublished data). Although predation pressure may be the selective force of most importance in this system, ‘predation environment’ is characterized by the presence (‘predator’) or absence (‘predator free’) of predators and a suite of other confounded environmental factors. For example, resource availability, stream gradient, and stream width may play an important role in determining life-history evolution and resulting morphology and are known to co-vary with presence or absence of predators in *B. rhabdophora* [56]. In this study, we consider ‘predation environment’ to be this suite of ecological features, which included either the presence or absence of piscivorous predators. *Brachyrhaphis roseni*, *B. terrabensis*, and *B. rhabdophora* typically occur in low velocity stream habitats (i.e., side-channels and pools found in small tributaries), although higher elevation sites (typical of *B. terrabensis* populations) tend to have steeper gradients and slightly faster stream velocities. *Brachyrhaphis terrabensis* primarily occurs in the same river drainages as *B. roseni*, although at higher elevations. *Brachyrhaphis roseni* habitat is characterized by low-elevation streams that are predator environments, while *B. terrabensis* occurs in predator free environments. *Brachyrhaphis rhabdophora* is found in both habitat types, predator free (typically high-elevation) and predator (typically low-elevation).

### Geometric Morphometric Analyses

We used a total of 802 fish in the geometric morphometric analysis (Appendix I): 211 *B. terrabensis* (predator free), 289 *B. roseni* (predator), and 302 *B. rhabdophora* (201 from predator, and 101 from predator free sites). For all sites, there were roughly equal numbers of males and females, and a representative sample of the range of size variation observed within each population. For each fish, we measured standard length (mm), and digitized thirteen biologically homologous landmarks (or semi-landmarks; Figure S1) on a lateral image of each fish (tpsDig; [72]). Landmarks were defined as: (1) anterior tip of the snout; (2), anterior extent of the eye; (3) semi-landmark midway between landmarks 1 and 4; (4) anterior insertion of the dorsal fin; (5) posterior insertion of the dorsal fin; (6) semi-landmark midway between landmarks 5 and 7; (7) dorsal origin of the caudal fin; (8) ventral origin of the caudal fin; (9) semi-landmark midway between landmarks 8 and 10; (10) posterior insertion of anal fin or gonopodium in males; (11) anterior insertion of the anal fin or gonopodium in males; and (12) semi-landmark midway between landmarks 11 and 13; (13) intersection of the operculum with the ventral outline of the body.

We summarized shape variation from digital landmarks into relative warps (i.e., principal components) using tpsRelw [73]. We used generalized Procrustes analysis [74] to remove all non-shape variation due to position, orientation, and scale of the specimens for each image. For sliding semi-landmarks we used the minimize d^2^ option in tpsRelw. Relative warps are defined as linear combinations of affine and non-affine shape components that describe some portion of the variation observed in the specimens [73]. We used the first 10 relative warps, which combined explained more than 96% of the shape variation, in subsequent analyses. By using only the top ten relative warps we effectively reduce the number of variables and account for the reduced dimensionality from use of sliding semi-landmarks. We analyzed the data using mixed model multivariate analysis of variance (MANOVA) in ASREML-R version 3.00 [75] within R (R Core Development Team 2010). Within each model, we included sampling site as a random factor to ensure that outlier sites did not drive the patterns we observed. Given that relative warps are orthogonal and ordered according to the amount of variation they explain, they can be treated as repeated measures with the use of an ‘index variable’ analogous to time in traditional repeated measures models. This method has been successfully employed in similar studies of shape variation in *B. rhabdophora* [6] and other livebearing fishes [76]. Thus, the order number of the relative warps (i.e. 1–10; reflecting the order of the warps but not the value) was treated as an index variable and included in the repeated statement for mixed model analyses. The use of the index variable arises out of mathematical necessity, and is crucial for this method to work and to interpret the results. It is the interaction of the main effect with the index variable that allows us to test the hypothesis that shape differs between groups on any one or any linear combination of relative warps. This is the same hypothesis tested in a standard MANOVA, but the index variable allows us to test this hypothesis in a mixed model framework. We tested each of our four hypotheses (detailed above) using these data.

To test for overall shape differences between predation environments (hypothesis 1), and for shape differences between predation environment and across sexes (hypothesis 3), we first tested for main effects and interactions of predation environment, sex, centroid size (a covariate; hereafter size), and index variable for the whole dataset (*N* = 802). Within each model, we included sampling site as a random factor to ensure that outlier sites did not drive the patterns we observed. Our initial global model estimated shape as ∼ index variable + species + sex + size + (index variable: species) + (index variable: sex) + (index variable: size) + (index variable: species: sex) + (index variable: species: size) + (index variable: sex: size) + (index variable: species: sex: size). We used model selection techniques (i.e., AIC) to determine if a reduced model (all possible models maintaining the fixed effects) resulted in a better model fit (i.e., lowest AIC score; [69], [77]). In our analysis, interactions between main effects and the index variable served as the most direct test of our hypotheses. Simple interactions of main effects are less informative because the interaction with the index variable tests for differences in shape on each of the relative warps independently, while simple interactions do not. If we do not consider the interaction with the index variable we are simply testing for differences among treatments when averaged across all relative warps. Relative warps are independent from each other (i.e., they explain different axes of variation); therefore the magnitude and direction of differences between levels of the main effects may vary differently and randomly across relative warps. Interactions with the index variable allow relative warps to vary independently (i.e., not to be considered as a whole) and thus allow the interaction to be significant even if the main effects alone, or their interactions, are not [6].

**Figure 2:**
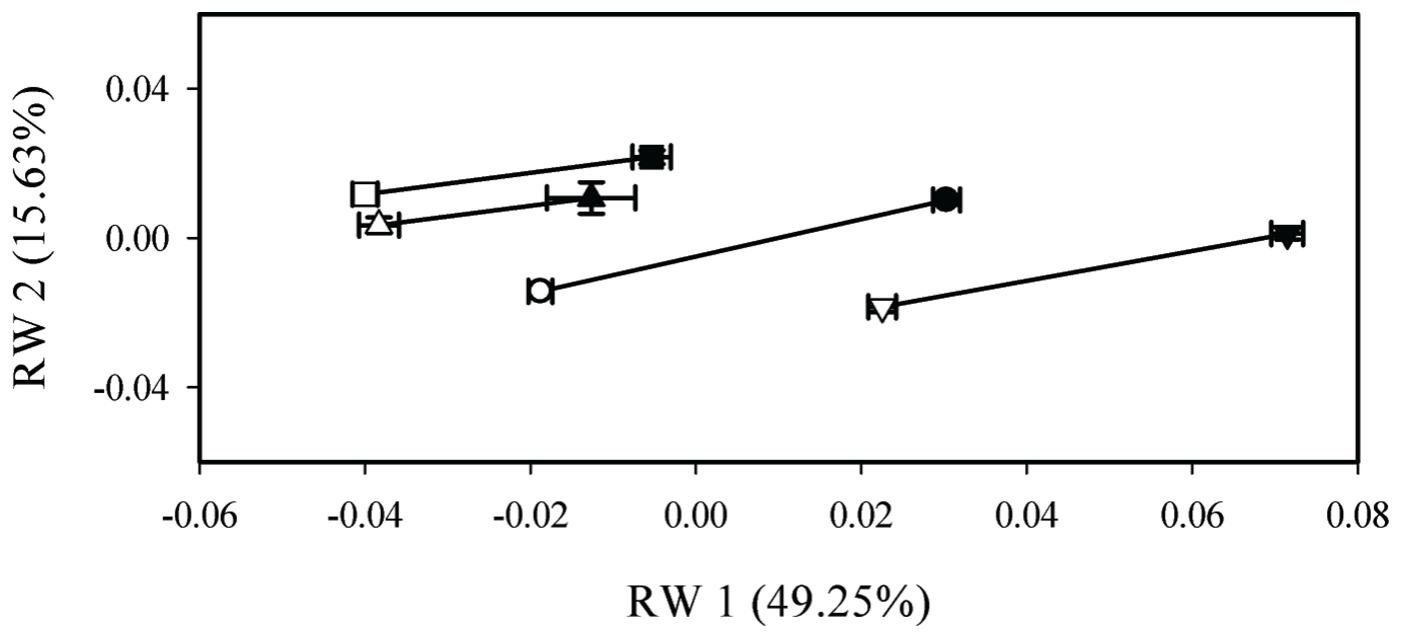
Least Square Means of Relative Warps. Graph of least square means of relative warp (RW) scores (±SE) for *Brachyrhaphis roseni* (•), *B. terrabensis* (▾), *B. rhabdophora* from predator environments (▪), and *B. rhabdophora* from predator free environments (▴). Filled symbols represent males, and open symbols represent females. Female body shape converges relative to male body shape in *B. roseni, B. terrabensis* and populations of *B. rhabdophora* from divergent predation environments.

Given that in both of our taxonomic contrasts we found a significant interaction between predation environment, sex, and the index variable in the MANOVA, we applied a phenotypic change vector analysis (PCVA; [78]–[80]) to determine the specific nature of the interaction to test for differences in shape changes between sexes. This analysis has been used previously and effectively in another *Brachyrhaphis* species [6]. The PCVA tests whether the significant interaction between main effects and the index variable resulted from differences in magnitude (*MD*) or direction (Θ) of morphological change. The PCVA tests magnitude and direction across all relative warps. Specifically, we used the PCVA to compare the amount and direction of sexual dimorphism between *B. roseni* and *B. terrabensis*, and between populations of *B. rhabdophora* from different predation environments. Here, we compared both size and direction of the phenotypic trajectories to test for differences in magnitude of sexual dimorphism and for different effects of predation on males and females (i.e., to determine if predation affects sexes differently), respectively. We conducted the PCVA using ASREML-R version 3.00 [75] within R (R Core Development Team 2010). We plotted LS means on the first two relative warp axes, which accounted for 64.36% of the shape variation, to visualize differences in magnitude and direction of shape change (Fig. 2).

To test for a difference in magnitude of variation between predation environment (hypothesis 2), and for differences between predation environment across sizes (hypothesis 4), we tested for main effects and interactions of species group (*B. roseni*/*B. terrabensis* and *B. rhabdophora* from divergent predation environments), predation environment, size, and index variable for each sex (males *N* = 278; females *N* = 523) using a mixed model MANOVA. We included location as a random variable in the model. Our full model estimated shape as  =  index variable + group + environment + size + (index variable: group) + (index variable: environment) + (index variable: size) + (index variable: group: environment) + (index variable: group: size) + (index variable: environment: size) + (index variable: group: environment: size). We used model selection techniques to determine if a reduced model resulted in a better model fit [69], [77]. Where the interaction of group, environment, and index variable was significant in the MANOVA, we applied the PCVA to determine whether the significant interaction between main effects and the index variable resulted from differences in *MD* or Θ of morphological change. Following significant interaction between size and the index variable, we generated thin-plate splines in tpsRegr [81] using centroid size and superimposed landmark coordinates to visualize shape variation along the centroid size axis.

## Results

### Effects of Predation Environment on Body Shape

Consistent with the predictions in our first hypothesis, we found that body shape differed between predation environments both within *Brachyrhaphis rhabdophora* and between *B. roseni* and *B. terrabensis*. The best-fit model estimated shape as ∼ index variable + species + sex + size + (index variable: species) + (index variable: sex) + (index variable: size) + (index variable: species: sex) + (index variable: species: size) + (index variable: sex: size) + (index variable: species: sex: size). Morphology differed significantly for the interaction of species group, predation environment, and index variable for both females and males (Table 1). Thus, we conducted a PCVA analysis to determine if the significant differences were caused by the magnitude of change, the direction/angle of change, or both for each sex (hypothesis 2). For females, the PCVA revealed that the magnitude of shape variation was greater in the *B. roseni/B. terrabensis* species group (*MD*  = 0.0348; P = 0.001); the trajectories also differed in orientation (θ = 80.14°; P = 0.001). Similarly, the PCVA revealed that the magnitude of shape variation in males was greater in the *B. roseni/B. terrabensis* species group (*MD*  = 0.0247; P = 0.001) and that the trajectories differed in orientation (θ = 81.80°; P = 0.002). Consistent with the predictions for our second hypothesis, greater morphological differentiation occurred between *B. roseni/B. terrabensis* than between populations of *B. rhabdophora* from different predation environments. Specifically, *B. rhabdophora* achieved 29% (females) and 47% (males) of the divergence present between *B. roseni/B. terrabensis*.

**Table 1:** Results of mixed-repeated-measures MANOVA testing for interactions between combinations of species-group, predation-environment, size and index-variable.

Effect	DF (fm)	F (f)	P (f)	F (m)	P (m)
Index variable	10	869.1	<0.001	1464.9	<0.001
Species group	1	78.4	<0.001	9.8	0.002
Predation	1	22.8	<0.001	0.2	0.649
Centroid size	1	16.2	<0.001	1.8	0.177
Species group × index variable	9	1756.8	<0.001	904.8	<0.001
Predation × index variable	9	697.5	<0.001	565.5	<0.001
Centroid size × index variable	9	517.0	<0.001	197.8	<0.001
Species group × predation × index variable	10	664.0	<0.001	118.6	<0.001

DF  =  degrees of freedom, f =  females, m =  males.

Morphology differed significantly for the interaction of predation environment, sex, and index variable (Table 2). Thus, we conducted a PCVA analysis to determine if the significant difference was caused by the magnitude of change, the direction/angle of change, or both. Summary statistics revealed that there was significant variation in the magnitude of sexually dimorphic shape change among the four taxa (Var_size_  = 0.0000977; P = 0.003) and significant variation in the direction of shape change (Var_orient_  = 257.57; P = 0.001). Within species groups, the magnitude of shape change was not significantly different; however, the magnitude of sexually dimorphic shape change was significantly greater in the *B. roseni/B. terrabensis* species group in all pairwise comparisons with the *B. rhabdophora* group (Table 3). The direction of shape change was significant in all pairwise comparisons (Table 3). For within species comparisons, the direction of shape change represented a convergence of shape in females, which was consistent with the predictions of our third hypothesis.

**Table 2:** Results of mixed-repeated-measures MANOVA examining shape variation and sexual dimorphism in *Brachyrhaphis*.

Effect	DF	F	P
Index variable	10	0.1	1
Species	3	50.8	<0.001
Sex	1	762.5	<0.001
Centroid size	1	3.4	0.06455
Species × index variable	27	4491.1	<0.001
Sex × index variable	9	1892.3	<0.001
Centroid size × index variable	9	663.2	<0.001
Species × sex × index variable	30	440.8	<0.001

DF  =  degrees of freedom.

**Table 3:** Statistical assessment of differences in trajectory size/ direction among trajectories characterizing sexual dimorphism in *Brachyrhaphis*.

Comparison	*MD*_1,2_	*P*_size_	*θ*_1,2_	*P*_θ_
1, 2	0.0019	0.583	**14.32**	**0.007**
1, 3	**0.0190**	**0.001**	**26.41**	**0.004**
1, 4	**0.0206**	**0.003**	**50.31**	**0.002**
2, 3	**0.0209**	**0.001**	**33.41**	**0.002**
2, 4	**0.0225**	**0.001**	**56.90**	**0.002**
3, 4	0.0016	0.808	**26.60**	**0.005**

*MD*_1,2_ =  trajectory size, *θ*_1,2_ =  trajectory direction, Taxa codes: 1 =  *Brachyrhaphis roseni*, 2 =  *B. terrabensis*, 3 =  *B. rhabdophora* from predator environments, and 4 =  *B. rhabdophora* from predator free environments. Significant differences generated empirically from 1,000 permutations are indicated in bold.

**Figure 3:**
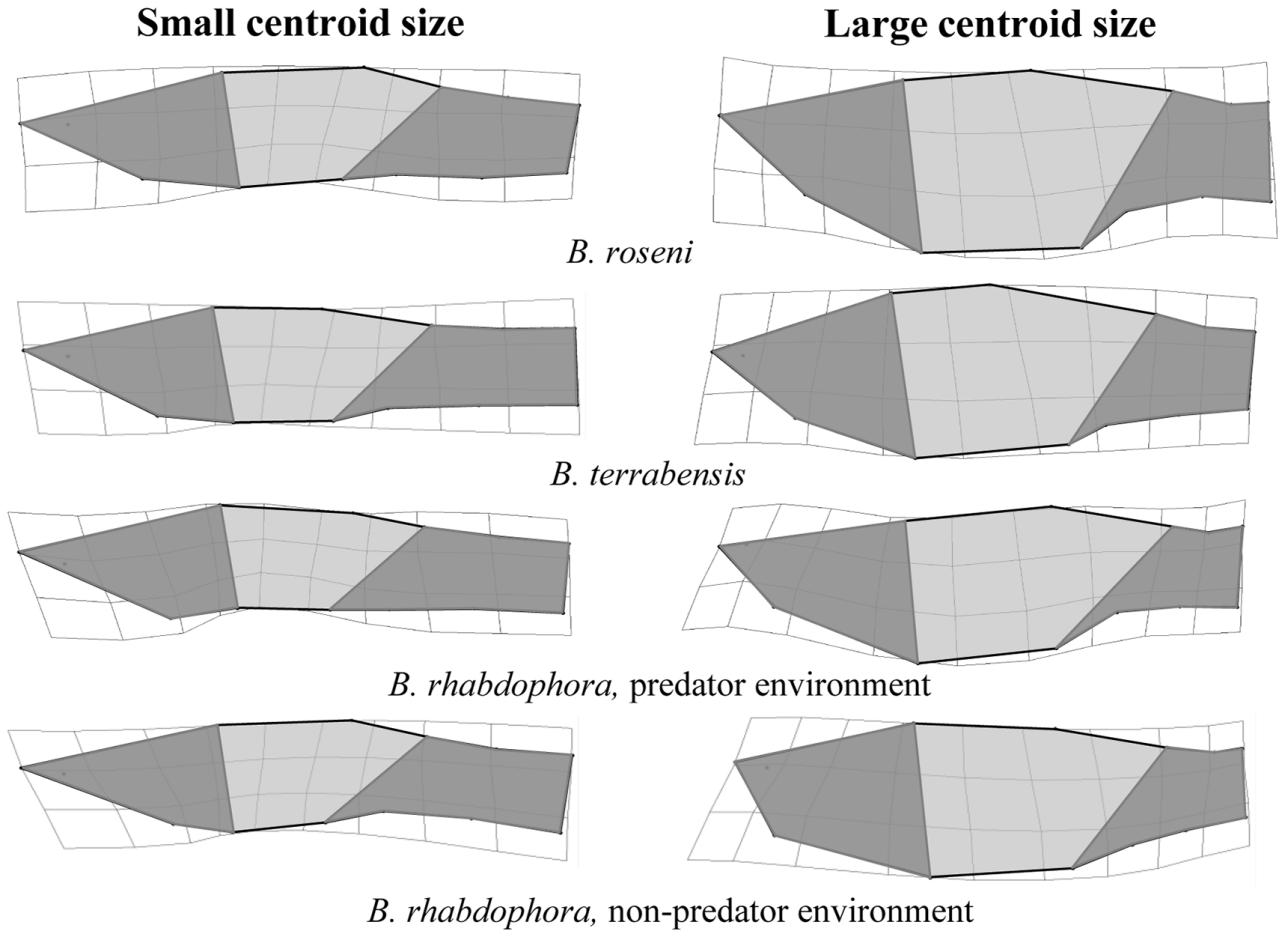
Morphological Divergence in Female *Brachyrhaphis*. Visualization of morphological divergence with centroid size in female *Brachyrhaphis roseni* (a), *B. terrabensis* (b), and *B. rhabdophora* from predator (c) and predator free (d) environments. Thin-plate spline transformations depict the end points of the centroid size axis (i.e. the smallest and largest individuals). Shaded regions are drawn to aid in interpretation. Note the increase in abdomen distension and decrease in caudle peduncle region in large females. Deformations are scaled to 3X to assist interpretation of the shape differences.

**Figure 4:**
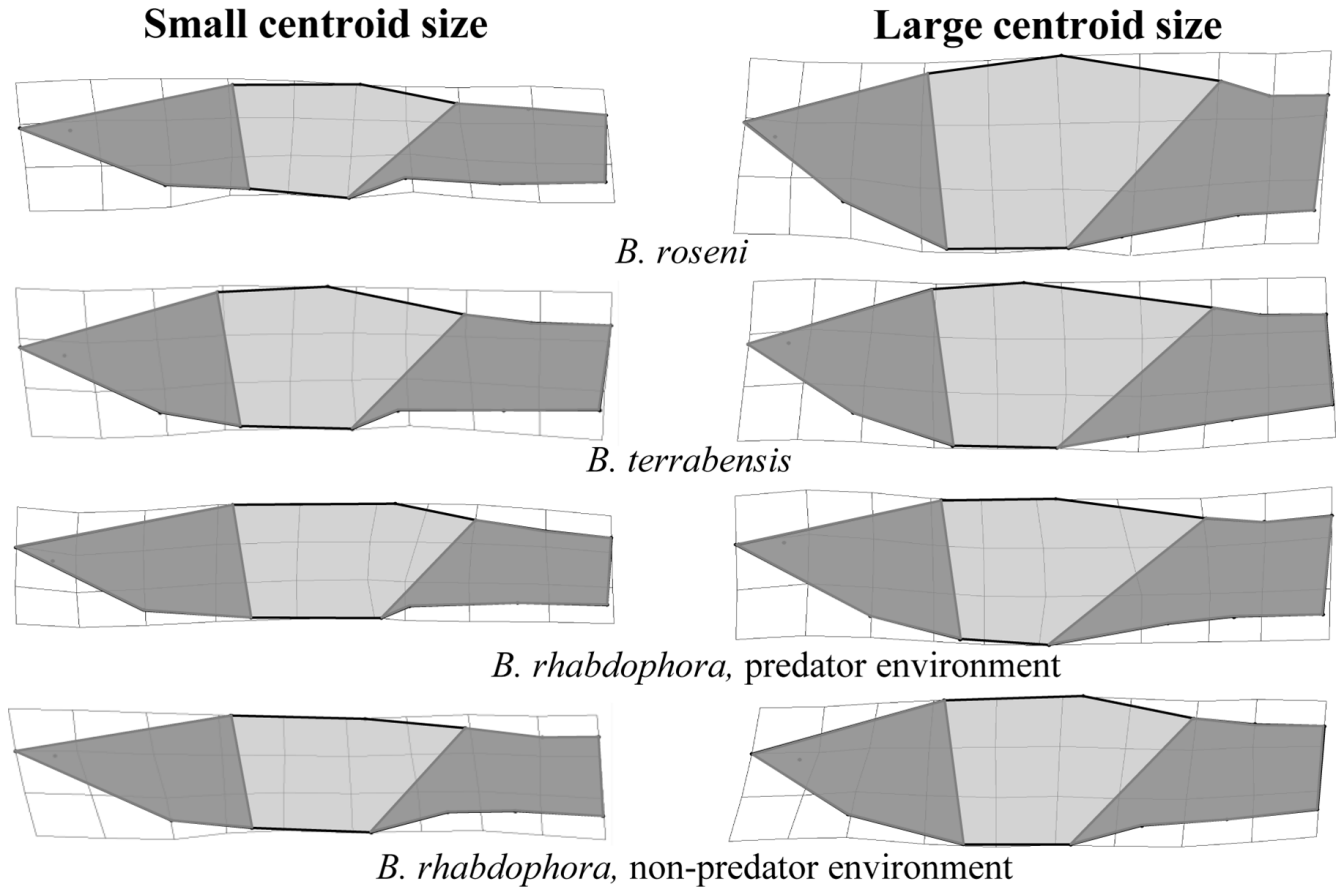
Morphological Divergence in Male *Brachyrhaphis*. Visualization of morphological divergence with centroid size in male *Brachyrhaphis roseni* (a), *B. terrabensis* (b), and *B. rhabdophora* from predator (c) and predator free (d) environments. Thin-plate spline transformations depict the end points of the centroid size axis (i.e. the smallest and largest individuals). Shaded regions are drawn to aid in interpretation. Note the shortening and deepening of the head region and the reduction in the caudle peduncle region in large males. Deformations are scaled to 3X to assist interpretation of the shape differences.

To determine how shape varies across size classes (hypothesis 4) in females (due to changes associated with pregnancy) and males (due to potential differences in mating strategies and ontogenetic effects), we generated thin-plate splines in tpsRegr [81] using centroid size and superimposed landmark coordinates to visualize shape variation along the centroid size axis in females (Fig. 3) and males (Fig. 4) of both species. We found that females showed a shift in morphology from small to large that was characterized by an increase in abdomen size and a decrease in caudal peduncle area. Adult males showed a shift in morphology from small to large that was characterized by a shortening and deepening of the head region and a reduction in the caudle peduncle region.

## Discussion

The principal objective of our study was to test for divergent morphologies driven by predation environment in *Brachyrhaphis* fishes at two taxonomic levels in two phylogenetically sister lineages, and determine how much variation occurs within populations and species that have evolved in similarly divergent selective regimes. We predicted that the divergent morphology observed between these species and populations would reflect body shape optimized for their native predation environment, although the magnitude of morphological divergence would be greater between *B. roseni* and *B. terrabensis* than between populations of *B. rhabdophora* from different predation environments. We also tested for differences in shape between sexes and across size classes, and predicted that shape optimization would differ across sex and size class according to potential differences in mating strategies or reproductive constraints.

### Parallel Morphological Evolution at Two Levels of Divergence

Our results strongly support divergent morphologies between *Brachyrhaphis roseni* and *B. terrabensis*, and between populations of *B. rhabdophora* from different predation environments as predicted by theory (Table 2; Fig. 2) [8], [51], [57]–[62], [82]. As predicted, individuals from predator environments showed a deeper caudal peduncle and a shallower anterior body/head than individuals from predator free environments. This pattern is strikingly similar to that observed in other poeciliids [8], [13], and strongly suggests that ‘predation environment’ is the principal driver of parallel patterns of shape variation between both sister species (*B. roseni* and *B. terrabensis*) and populations within a species (*B. rhabdophora*). Importantly, although our results suggest that both male and female body shape was significantly more divergent (i.e., more pronounced) between *B. roseni* and *B. terrabensis* than between *B. rhabdophora* populations from different predation environments (Fig. 2), 47% (males) and 29% (females) of the variation in body shape was already present between populations of *B. rhabdophora*. Therefore, although sister species *B. roseni* and *B. terrabensis* are clearly at a point of greater divergence (i.e., phylogenetically but also potentially ecologically), both taxon pairs are on a similar evolutionary trajectory and *B. rhabdophora* has already reached a substantial level o cf evolutionary diversification. Intraspecific evolutionary divergence of this type has been noted in a variety of poeciliid fishes for several different traits [13], [39], [40], [46]–[49]. Interestingly, we found that in *B. rhabdophora* divergence in male morphology was greater than divergence in female morphology, at least relative to variation noted between *B. roseni and B. terrabensis*. This pattern of males evolving more rapidly than females has previously been noted in guppies in work that focused on life history traits [83]. Following an introduction experiment, which involved transplanting populations from high-predation to low-predation sites, evolution of male life-history traits was significantly more rapid than female life-history traits [83]. This finding was largely attributed to a difference in heritability, possibly associated with Y chromosome-linked traits [83]. The pattern observed in *Brachyrhaphis* suggests that female body shape is less variable, perhaps due to constraints associated with pregnancy (see below). The fact that male *B. rhabdophora* have achieved a greater amount of divergence relative to females may be due to greater existing variation in male body shape. One possible explanation is that males that employ alternative mating strategies have evolved different morphologies to accommodate these strategies (see below). If males of different sizes do in fact tend to adopt alternative mating strategies, it would be likely that greater genetic variance would occur in males relative to females, possibly contributing to the greater differentiation achieved in male *B. rhabdophora* relative to female *B. rhabdophora*. Overall, we see four possible explanations for why greater divergence occurs between *B. roseni* and *B. terrabensis* than occurs within *B. rhabdophora*, although we did not explicitly test any of these hypotheses, and only briefly state them here. First, the time since *B. roseni* and *B. terrabensis* diverged could be greater than the time since populations of *B. rhabdophora* from predator and predator free environments. Second, *B. roseni* and *B. terrabensis* could be experiencing stronger divergent selection than *B. rhabdophora*. Third, populations of *B. rhabdophora* and sister species *B. roseni-B. terrabensis* could be experiencing differences in the balance between selection and gene flow. And finally, greater heritable variation could be present between *B. roseni* and *B. terrabensis* relative to *B. rhabdophora*. These hypotheses should be tested further to determine the exact nature of this difference in relative morphological divergence.

The idea that *Brachyrhaphis roseni* and *B. terrabensis* are sister taxa that occur in the same drainages but in different predation regimes suggests the possibility that divergent natural selection has driven and maintains reproductive isolation between these two species. Numerous lines of evidence suggest that the most recent common ancestor of this species pair likely occurred across a range of predation habitats within the drainages where *B. roseni* and *B. terrabensis* are currently found, a pattern strikingly similar to that found in congenerics *B. rhabdophora* [24], [25], [43], [46], [56] and *B. episcopi* [23], [42], [84]. For example, multiple recently diverged populations of *B. rhabdophora* have evolved life-history phenotypes that are adaptive for their specific predation environments [24], [25], [43], [46], [56]. *Brachyrhaphis roseni* and *B. terrabensis* have evolved nearly identical, although more pronounced, life-history phenotypes as a result of divergent selection regimes (Belk et al., in review). Likewise, our results suggest that body shape evolution is also occurring in parallel, with similar but more pronounced divergence in *B. roseni* and *B. terrabensis* than is found in *B. rhabdophora*. This begs the question: have similarly divergent selection regimes also driven the evolution of reproductive isolation in parallel? Previous studies suggest that body shape plays a key role in mate choice in other livebearing fish, and that individuals prefer as mates those who have a body shape optimized for selection regimes similar to their own [7]. If this holds true in *Brachyrhaphis*, it is likely that reproductive isolation due to assortative mating for body shape may already occur between populations of *B. rhabdophora*, and is even stronger between *B. roseni* and *B. terrabensis*. Studies in our lab are currently underway to test these predictions.

### Reproductive Constraints on Morphological Evolution

Although shape varied between *B. roseni* and *B. terrabensis*, and between populations of *B. rhabdophora* from different predation environments as predicted (hypothesis 1), the degree of variation was not equal across sexes (hypothesis 3). As predicted, both male and female diverged as a function of predation environment; however, divergence in female shape was less than divergence in male shape (Fig. 2). One explanation for this is that *Brachyrhaphis* are livebearing fishes with a female body shape constrained by pregnancy [6], regardless of predation environment. Hence, immature females from different predation environments might initially differ in body shape, but these differences go away once females become pregnant. This difference is predicted by a tradeoff that occurs between reproduction and fast-start swimming performance (i.e., pregnant females have reduced fast-start speeds), as observed in another poeciliid species [6], [37]. This observation of female shape convergence also illuminates previous patterns observed regarding mortality rates in the closely related *B. rhabdophora* [25]. Johnson and Zuniga-Vega (2009) showed that differential mortality rates drive life-history evolution in *B. rhabdophora* (i.e., higher survivorship in predator free environments than in predator environments), and that in predator environments mortality rates were relatively constant across size classes until individuals reached the largest size class where mortality increases. This pattern is reversed in predator free environments (i.e., survivorship increases in the largest size class). If convergence in body shape coincides with divergent mortality rates as size increases, then our data suggest that *B. roseni* and *B. terrabensis* should also be experiencing differences in size-specific mortality rates. A possible explanation is the negative impact that pregnancy may have on fast start swimming performance (useful in predator environments) as seen in related poeciliid fish [37].

### Morphological Evolution across Size Classes: Role of Sexual Selection and Alternative Mating Strategies?

In addition to finding gross differences in morphology between predation environments, we found evidence that shape did not vary consistently among size classes of adult females (Fig. 3) and males (Fig. 4) of all *Brachyrhaphis* species studied. In other words, we found allometric differences in shape among size classes in each taxon. We predicted that shape would not vary consistently across sizes (i.e., as individuals mature and grow) because of the potential variation in male reproductive strategy across size classes in *Brachyrhaphis*, and differences in female reproductive allocation at different sizes. As adult females increase in size, the predominant shape change that occurs is a relative increase in abdomen size and a resulting relative decrease in the caudal peduncle region. This finding complements Wesner et al. (2011), who found that late in pregnancy, female body shape converges due to constraints of pregnancy on body shape. The patterns observed between female *B. roseni* and *B. terrabensis*, and *B. rhabdophora* from different predation environments, is remarkable similar.

The pattern of shape change with size in mature males follows a different pattern, potentially consistent with different reproductive strategies between small and large males (i.e., sneaker males vs. displaying males) in each species. Patterns of shape variation with size observed in males of *B. roseni, B. terrabensis*, and *B. rhabdophora* are consistent with shapes that are optimized for behaviors associated with reproductive mode; within taxonomic units, small males had a body shape that facilitated burst swimming more than large males (e.g., more streamlined with a more robust caudal peduncle), who demonstrated a body shape that was more conducive to endurance swimming necessary for displaying behaviors (i.e., deeper anterior body/head region with a relatively shallow peduncle) [12]–[14], [51], [55]. The size at which a male reaches maturity has a large effect on mode of reproduction in numerous livebearing fish [85]–[87] because males typically do not grow after maturing. Relatively smaller males (“sneakers”) often rely on forced copulations (i.e., coercion) rather than courting females to win mates, although the degree to which this pattern holds is highly species specific; mating strategy is context dependent [82], [86]–[90] in some species (i.e., relative size determines mating strategy), while in others mating strategy is genetically based and not plastic [86], [87], [91]. Preliminary observations suggest that small *Brachyrhaphis* males tend to sneak (especially in the presence of larger males), while larger males devote more of their reproductive efforts to displaying to win mates (personal observation). Although species-specific variation in mating strategies exists, some patterns can be generalized. Forced copulation generally relies on short swimming bursts [86], [87] that allow the male to copulate with a female before she can defend herself and potentially injure the male. Alternatively, relatively large males adopt larger, showier features and often rely on a courting strategy of reproduction (i.e., coaxing) [86], [87]. Displaying males are often required to swim alongside a female until she concedes copulation (personal observation). We hypothesize that this mode of reproduction is likely optimized by a more fusiform body shape that allows the male to have greater swimming endurance during courtship. Just as livebearing reproduction interacts antagonistically with predation environment in generating female morphology (i.e., pregnancy constraints and resulting swimming performance trade-offs), reproductive mode and predation environment may exert opposing selective pressures on body shape in males. We propose that the nearly identical patterns we observed at both taxonomic levels we tested here suggests that selection could favor different body forms that may be associated with reproductive roles and mating strategies, and that the potential adaptive nature of different behaviors is paralleled by morphological divergence. Our findings, although they do not provide conclusive evidence in support of this hypothesis, highlight a gap in our knowledge related to the role of morphology in alternative mating strategies. Future work should focus on determining how body shape and size interplay with mating strategies, whether genetically determined or plastic.

## Conclusions

In conclusion, sister taxa *Brachyrhaphis roseni* and *B. terrabensis* differed dramatically in body shape and the differences observed correspond to divergent predation regimes that favor different body shapes. *Brachyrhaphis rhabdophora* from different predation environments also differ as predicted by predation environment, and these differences are parallel, although less exaggerated, to those observed between *B. roseni* and *B. terrabensis*. Our study provides evidence that evolution acts in a predictable manner when similar selection pressures are at work by showing that body shape evolution follows dramatically similar trajectories at multiple levels of divergence (i.e., both between and within species). We also conclude that shape appears to be optimized differently in males and females, and across a range of sizes, and that these differences may correspond to reproductive roles and mating strategies, respectively. The fact that closely related species in geographic proximity and similar selective environments have evolved nearly identical morphological characteristics is strong evidence that evolution acts in a predictable manner, and provides a framework for future studies on speciation in this unique system.

## Supporting Information

Figure S1 **Geometric morphometric landmarks.** Landmark locations used for geometric morphometric analyses on *Brachyrhaphis roseni*, *B. terrabensis*, and *B. rhabdophora.*
(DOCX)

Table S1 **Geometric morphometric population data.** Population data for samples used in the geometric morphometric portion of this study, including total N, drainage and country of origin, and coordinates.
(DOCX)

Table S2 **Genetic population data.** Population data for samples used in the pairwise analyses of genetic distance, including total sample size (N), drainage and country of origin, and coordinates. All sequences are deposited on Genbank under accession numbers KJ081551 – KJ081609.
(DOCX)

Table S3 **Genetic distance comparisons within** ***Brachyrhaphis rhabdophora.*** Pairwise genetic distances based on 1140 base pairs of cytochrome *b* (plus ∼65 bp of the downstream gene) for *Brachyrhaphis rhabdophora* from high- (HP) and low-predation (LP) environments. Raw pairwise differences are presented above the diagonal, and adjusted pairwise differences using TrN+G model of evolution are presented below the diagonal.
(DOCX)

Table S4 **Genetic distance comparisons between** ***Brachyrhaphis roseni*** **and** ***B. terrabensis.*** Pairwise genetic distances based on 1140 base pairs of cytochrome *b* (plus ∼65 bp of the downstream gene) for *Brachyrhaphis roseni* and *B. terrabensis*. Raw pairwise differences are presented above the diagonal, and adjusted pairwise differences using TrN+G model of evolution are presented below the diagonal. Population abbreviations for drainage of origin are as follows: Rio Chiriquí (Ch.); Rio Chiriquí Viejo (CV); and Rio Coto (C). Two populations of *B. terrabensis* were taken from the Rio Chiriquí Viejo drainage, and are designated with subscripts representing their country of origin (CV_CR_ and CV_P_ for Costa Rica and Panama, respectively).
(DOCX)
