# Supplementary material for: Morphological Divergence Driven by Predation Environment within and between Species of Brachyrhaphis Fishes
Source: PLoS One. 2014 Feb 26;9(2):e90274. doi: 10.1371/journal.pone.0090274 (PMC3936007; doi:10.1371/journal.pone.0090274)

**Figure S1. Geometric morphometric landmarks.** Landmark locations used for geometric morphometric analyses on *Brachyrhaphis roseni*, *B. terrabensis,* and *B. rhabdophora.*


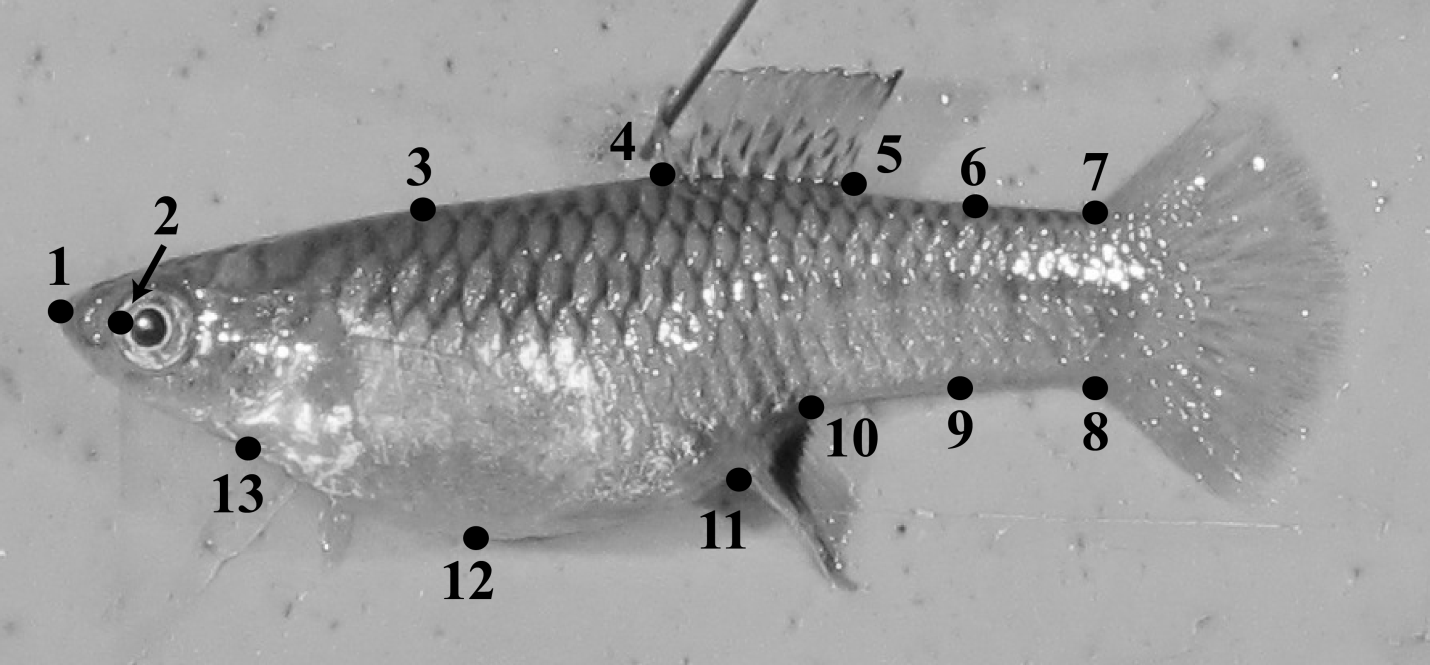

Supplement: Figure S1 — Geometric morphometric landmarks. Landmark locations used for geometric morphometric analyses on Brachyrhaphis roseni, B. terrabensis, and B. rhabdophora. (DOCX) [file pone.0090274.s001.docx]
