# Supplementary material for: Morphological Divergence Driven by Predation Environment within and between Species of Brachyrhaphis Fishes
Source: PLoS One. 2014 Feb 26;9(2):e90274. doi: 10.1371/journal.pone.0090274 (PMC3936007; doi:10.1371/journal.pone.0090274)
Supplement: Table S1 — Geometric morphometric population data. Population data for samples used in the geometric morphometric portion of this study, including total N, drainage and country of origin, and coordinates. (DOCX) [file pone.0090274.s002.docx]

**Table S1. Geometric morphometric population data.** Population data for samples used in the geometric morphometric portion of this study, including total N, drainage and country of origin, and coordinates.

| **Species** | **Total (N)** | **Drainage** | **Country** | **Coordinates** |
| --- | --- | --- | --- | --- |
| *B. roseni* | 41 | Rio Chiriquí Viejo | Panama | N 8.5184, W 82.7115 |
| *B. roseni* | 38 | Rio Chiriquí Viejo | Panama | N 8.53371, W 82.6734 |
| *B. roseni* | 132 | Rio Chiriquí | Panama | N 8.4251, W 82.4176 |
| *B. roseni* | 40 | Rio Chiriquí | Panama | N 8.4304, W 82.4209 |
| *B. roseni* | 38 | Rio Coto | Costa Rica | N 8.6551, W 82.9463 |
| *B. terrabensis* | 40 | Rio Chiriquí Viejo | Costa Rica | N 8.8802, W 82.8571 |
| *B. terrabensis* | 69 | Rio Chiriquí Viejo | Panama | N 8.7924, W 82.6566 |
| *B. terrabensis* | 21 | Rio Chiriquí Viejo | Panama | N 8.8294, W 82.7154 |
| *B. terrabensis* | 33 | Rio Chiriquí Viejo | Panama | N 8.7183, W 82.8118 |
| *B. terrabensis* | 49 | Rio Chiriquí | Panama | N 8.6609, W 82.5206 |
| *B. rhabdophora* high-predation | 114 | Rio Jesus Maria | Costa Rica | N 9.9604, W 84.6066 |
| *B. rhabdophora* high-predation | 43 | Rio San Rafael | Costa Rica | N 9.9844, W84.6252 |
| *B. rhabdophora* high-predation | 44 | Rio Piedras | Costa Rica | N 10.5297, W 85.2809 |
| *B. rhabdophora* no-predation | 65 | Quebrada Grande | Costa Rica | N 10.4415, W 84.9877 |
| *B. rhabdophora* no-predation | 35 | Rio Machuca | Costa Rica | N 9.9632, W 84.4911 |
