# Supplementary material for: Morphological Divergence Driven by Predation Environment within and between Species of Brachyrhaphis Fishes
Source: PLoS One. 2014 Feb 26;9(2):e90274. doi: 10.1371/journal.pone.0090274 (PMC3936007; doi:10.1371/journal.pone.0090274)
Supplement: Table S2 — Genetic population data. Population data for samples used in the pairwise analyses of genetic distance, including total sample size (N), drainage and country of origin, and coordinates. All sequences are deposited on Genbank under accession numbers KJ081551 – KJ081609. (DOCX) [file pone.0090274.s003.docx]

**Table S2. Genetic population data.** Population data for samples used in the pairwise analyses of genetic distance, including total sample size (N), drainage and country of origin, and coordinates. All sequences are deposited on Genbank under accession numbers KJ081551 - KJ081609.

| **Species** | **Total (N)** | **Drainage** | **Country** | **Coordinates** |
| --- | --- | --- | --- | --- |
| *B. roseni* | 4 | Rio Chiriquí | Panama | N 8.4251, W 82.4176 |
| *B. roseni* | 8 | Rio Chiriquí Viejo | Panama | N 8.5184, W 82.7115 |
| *B. roseni* | 3 | Rio Coto | Costa Rica | N 8.6551, W 82.9463 |
| *B. terrabensis* | 8 | Rio Chiriquí | Panama | N 8.6609, W 82.5206 |
| *B. terrabensis* | 7 | Rio Chiriquí Viejo | Panama | N 8.7183, W 82.8118 |
| *B. terrabensis* | 2 | Rio Chiriquí Viejo | Costa Rica | N 8.8802, W 82.8571 |
| *B. rhabdophora* high-predation | 6 | Rio Javilla | Costa Rica | N 10.4024, W 85.0755 |
| *B. rhabdophora* high-predation | 5 | Rio Ciruelas (lower) | Costa Rica | N 10.0603, W 84.7586 |
| *B. rhabdophora* no-predation | 7 | Quebrada Grande | Costa Rica | N 10.4415, W 84.9877 |
| *B. rhabdophora* no-predation | 6 | Rio Ciruelas (upper) | Costa Rica | N 10.1008, W 84.7250 |
