# Supplementary material for: Morphological Divergence Driven by Predation Environment within and between Species of Brachyrhaphis Fishes
Source: PLoS One. 2014 Feb 26;9(2):e90274. doi: 10.1371/journal.pone.0090274 (PMC3936007; doi:10.1371/journal.pone.0090274)
Supplement: Table S3 — Genetic distance comparisons within Brachyrhaphis rhabdophora. Pairwise genetic distances based on 1140 base pairs of cytochrome b (plus ∼65 bp of the downstream gene) for Brachyrhaphis rhabdophora from high- (HP) and low-predation (LP) environments. Raw pairwise differences are presented above the diagonal, and adjusted pairwise differences using TrN+G model of evolution are presented below the diagonal. (DOCX) [file pone.0090274.s004.docx]

**Table S3. Genetic distance comparisons within *Brachyrhaphis rhabdophora.*** Pairwise genetic distances based on 1140 base pairs of cytochrome *b* (plus ~65 bp of the downstream gene) for *Brachyrhaphis rhabdophora* from high- (HP) and low-predation (LP) environments. Raw pairwise differences are presented above the diagonal, and adjusted pairwise differences using TrN+G model of evolution are presented below the diagonal.

|  | **Grande (LP)** | **Javilla (HP)** | **Lower Ciruelas (HP)** | **Upper Ciruelas (LP)** |
| --- | --- | --- | --- | --- |
| **Grande (LP)** | - | 0.003 | 0.008 | 0.003 |
| **Javilla (HP)** | 0.003 | - | 0.007 | 0.003 |
| **Lower Ciruelas (HP)** | 0.009 | 0.008 | - | 0.004 |
| **Upper Ciruelas (LP)** | 0.003 | 0.003 | 0.005 | - |
