# Supplementary material for: Morphological Divergence Driven by Predation Environment within and between Species of Brachyrhaphis Fishes
Source: PLoS One. 2014 Feb 26;9(2):e90274. doi: 10.1371/journal.pone.0090274 (PMC3936007; doi:10.1371/journal.pone.0090274)
Supplement: Table S4 — Genetic distance comparisons between Brachyrhaphis roseni and B. terrabensis. Pairwise genetic distances based on 1140 base pairs of cytochrome b (plus ∼65 bp of the downstream gene) for Brachyrhaphis roseni and B. terrabensis. Raw pairwise differences are presented above the diagonal, and adjusted pairwise differences using TrN+G model of evolution are presented below the diagonal. Population abbreviations for drainage of origin are as follows: Rio Chiriquí (Ch.); Rio Chiriquí Viejo (CV); and Rio Coto (C). Two populations of B. terrabensis were taken from the Rio Chiriquí Viejo drainage, and are designated with subscripts representing their country of origin (CVCR and CVP for Costa Rica and Panama, respectively). (DOCX) [file pone.0090274.s005.docx]

**Table S4. Genetic distance comparisons between *Brachyrhaphis roseni* and *B. terrabensis.*** Pairwise genetic distances based on 1140 base pairs of cytochrome *b* (plus ~65 bp of the downstream gene) for *Brachyrhaphis roseni* and *B. terrabensis*. Raw pairwise differences are presented above the diagonal, and adjusted pairwise differences using TrN+G model of evolution are presented below the diagonal. Population abbreviations for drainage of origin are as follows: Rio Chiriquí (Ch.); Rio Chiriquí Viejo (CV); and Rio Coto (C). Two populations of *B. terrabensis* were taken from the Rio Chiriquí Viejo drainage, and are designated with subscripts representing their country of origin (CV_CR_ and CV_P_ for Costa Rica and Panama, respectively).

|  | ***B. roseni –* Ch.** | ***B. roseni -*CV** | ***B. roseni -* C** | ***B. terrabensis –* Ch.** | ***B. terrabensis –* CV_P_** | ***B. terrabensis –* CV_CR_** |
| --- | --- | --- | --- | --- | --- | --- |
| ***B. roseni –* Ch.** | - | 0.006 | 0.014 | 0.036 | 0.043 | 0.042 |
| ***B. roseni -*CV** | 0.006 | - | 0.010 | 0.034 | 0.043 | 0.042 |
| ***B. roseni -* C** | 0.014 | 0.010 | - | 0.037 | 0.045 | 0.046 |
| ***B. terrabensis –* Ch.** | 0.039 | 0.036 | 0.040 | - | 0.026 | 0.025 |
| ***B. terrabensis –* CV_P_** | 0.047 | 0.047 | 0.049 | 0.028 | - | 0.001 |
| ***B. terrabensis –* CV_CR_** | 0.045 | 0.045 | 0.050 | 0.027 | 0.001 | - |
